# Development of a bispecific immune engager using a recombinant malaria protein

**DOI:** 10.1038/s41419-021-03611-0

**Published:** 2021-04-06

**Authors:** Mie A. Nordmaj, Morgan E. Roberts, Emilie S. Sachse, Robert Dagil, Anne Poder Andersen, Nanna Skeltved, Kaare V. Grunddal, Sayit Mahmut Erdoğan, Swati Choudhary, Tobias Gustsavsson, Maj Sofie Ørum-Madsen, Igor Moskalev, Weihua Tian, Zhang Yang, Thomas M. Clausen, Thor G. Theander, Mads Daugaard, Morten A. Nielsen, Ali Salanti

**Affiliations:** 1 Centre for Medical Parasitology at Department of Immunology and Microbiology, University of Copenhagen, Copenhagen, Denmark; 2 Department of Infectious Diseases, Copenhagen University Hospital, Copenhagen, Denmark; 3 Vancouver Prostate Centre, Department of Urologic Sciences, University of British Columbia, Vancouver, BC Canada; 4 Copenhagen Center for Glycomics, Departments of Cellular and Molecular Medicine, Faculty of Health Sciences, University of Copenhagen, Copenhagen, Denmark; 5 Department of Cellular and Molecular Medicine, University of California, San Diego, La Jolla, CA 92093 United States

**Keywords:** Drug development, Preclinical research

## Abstract

As an immune evasion and survival strategy, the *Plasmodium falciparum* malaria parasite has evolved a protein named VAR2CSA. This protein mediates sequestration of infected red blood cells in the placenta through the interaction with a unique carbohydrate abundantly and exclusively present in the placenta. Cancer cells were found to share the same expression of this distinct carbohydrate, termed oncofetal chondroitin sulfate on their surface. In this study we have used a protein conjugation system to produce a bispecific immune engager, V-aCD3, based on recombinant VAR2CSA as the cancer targeting moiety and an anti-CD3 single-chain variable fragment linked to a single-chain Fc as the immune engager. Conjugation of these two proteins resulted in a single functional moiety that induced immune mediated killing of a broad range of cancer cells in vitro and facilitated tumor arrest in an orthotopic bladder cancer xenograft model.

## Introduction

Cancer immunotherapy in the form of bispecific antibodies has been widely explored in a variety of formats, and has shown great promise across various tumor types^1–3^. Bispecific antibodies that engage CD3 on T cells and bind a tumor-specific marker on cancer cells can direct and activate T cells to the tumor regardless of T-cell receptor specificity and antigen presentation^4^. The efficacy of CD3-engaging bispecific antibodies has been demonstrated in several clinical trials^5–7^. However, the technology relies heavily on the identification of tumor targets that are absent in healthy human tissues to avoid off-target effects. Chondroitin Sulfate (CS) is a glycosaminoglycan (GAG). GAGs are long linear carbohydrates, made up of repeated disaccharide units attached to a serine in the protein core of proteoglycans^8^. CS is abundantly expressed throughout the human body but exhibits large variation in both chain length and disaccharide modifications, such as sulfation patterns^9^. It is well known that the expression and composition of GAGs change in cancer^9–12^. We recently discovered that a broad range of cancer cells express a distinct type of CS termed oncofetal CS (ofCS), which are also present on the rapidly dividing trophoblast cells in the placenta^13^. Red blood cells infected with the *Plasmodium falciparum* malaria parasite sequester in the placenta through the expression of a parasite-derived protein, VAR2CSA, that binds ofCS^14,15^. A recombinant subunit of VAR2CSA (rVAR2) binds with high affinity and specificity to ofCS expressed on the surface of cancer cells and in the tumor extracellular matrix, but exhibits minimal binding to CS expressed in healthy tissue besides the placenta^13,15^. Thus, cancer cells can be targeted using rVAR2^13,16–19^.

Anti-CD3 is the effector moiety of Catumaxomab^20,21^ and Blinatumomab^22^, in which the tumor-targeting moieties bind EpCAM and CD19, respectively. More recently, reports demonstrate clinical efficacy with CD3 bispecific antibodies targeting solid tumors in colorectal and prostate cancer^23–25^. Anti-CD3-engaging molecules are important effector components of several other bispecific anticancer drugs currently in clinical development^3^. These compounds bind cancer cells with the targeting moiety, and activate T cells by binding and engaging CD3. This results in T-cell activation through CD3/T-cell receptor signaling, and subsequent killing of the cancer cells^26^.

Here, we show proof of concept for targeting ofCS in immunotherapy using a novel bispecific molecule, V-aCD3, which employs recombinant rVAR2 as the cancer binding entity and the well-established anti-CD3 and single-chain murine IgG2b Fc molecule (scFv-sFc; clone OKT3) to bind immune cells. We utilized the SpyCather/SpyTag split protein to generate V-aCD3. The system relies on the spontaneous formation of an isopeptide bond in a protein from a *Streptococcus pyogenes* protein and a small peptide tag derived from the same protein. By coupling each of these two components to two different molecular entities, these can be mixed and attached to each other by a covalent bond^27,28^. Using such a modular approach also allows us to examine the effect of each component (i.e., rVAR2 and aCD3) and compare to the conjugated bispecific protein. Given the high specificity and broad tumor-targeting potential of rVAR2-based immunotherapies this work demonstrates that targeting ofCS has the potential to benefit patients with a wide variety of tumor types, including those that currently lack specific targeting strategies.

## Results

### Design of the bispecific V-aCD3 molecule

**Fig. 1:**
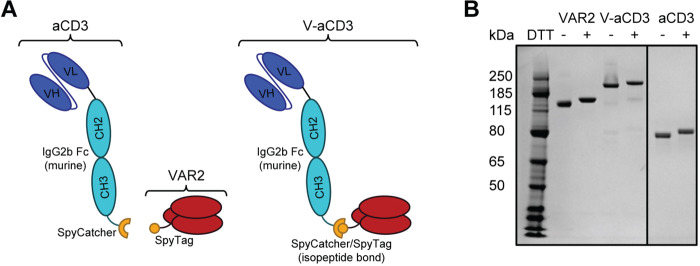
Design and purity of V-aCD3. **A** Schematic figure of the construction and assembly of V-aCD3. A single-chain anti-CD3 antibody (scFv (OKT3)-Fc (murine IgG2b)) was produced with a SpyCatcher domain, which spontaneously forms a covalent bond with SpyTagged VAR2. **B** SDS-PAGE showing recombinant rVAR2 (lane 2 nonreduced, lane 3 reduced), V-aCD3 (lane 4 nonreduced, lane 5 denatured), and anti-CD3 (lane 6 nonreduced, lane 7 reduced).

A bispecific molecule was generated using a technology in which a short peptide (SpyTag) spontaneously forms a covalent peptide bond to a protein partner (SpyCatcher)^29,30^. The SpyCatcher sequence was genetically fused to the C-terminus of the murine IgG2b Fc domain followed by anti-human CD3, and expressed in CHO cells. The SpyTag was genetically fused to the N-terminus of the ofCS binding region of rVAR2^15^ and expressed in SHuffle *Escherichia coli* (Fig. 1A). The bispecific immune engager (V-aCD3) was formulated by combining the two recombinant proteins in a 1:1 molar ratio. Analysis by SDS-PAGE indicated highly efficient conjugation of rVAR2(121 kD) and anti-CD3 scFv-Fc (65 kD) generating V-aCD3 with the expected molecular size of 186 kD (Fig. 1B). Analyses of the protein under nonreducing conditions show that the highly cysteine rich protein does not form interprotein disulfide bonds.

### V-aCD3 maintains the binding and specificity of rVAR2 to oncofetal chondroitin sulfate

**Fig. 2:**
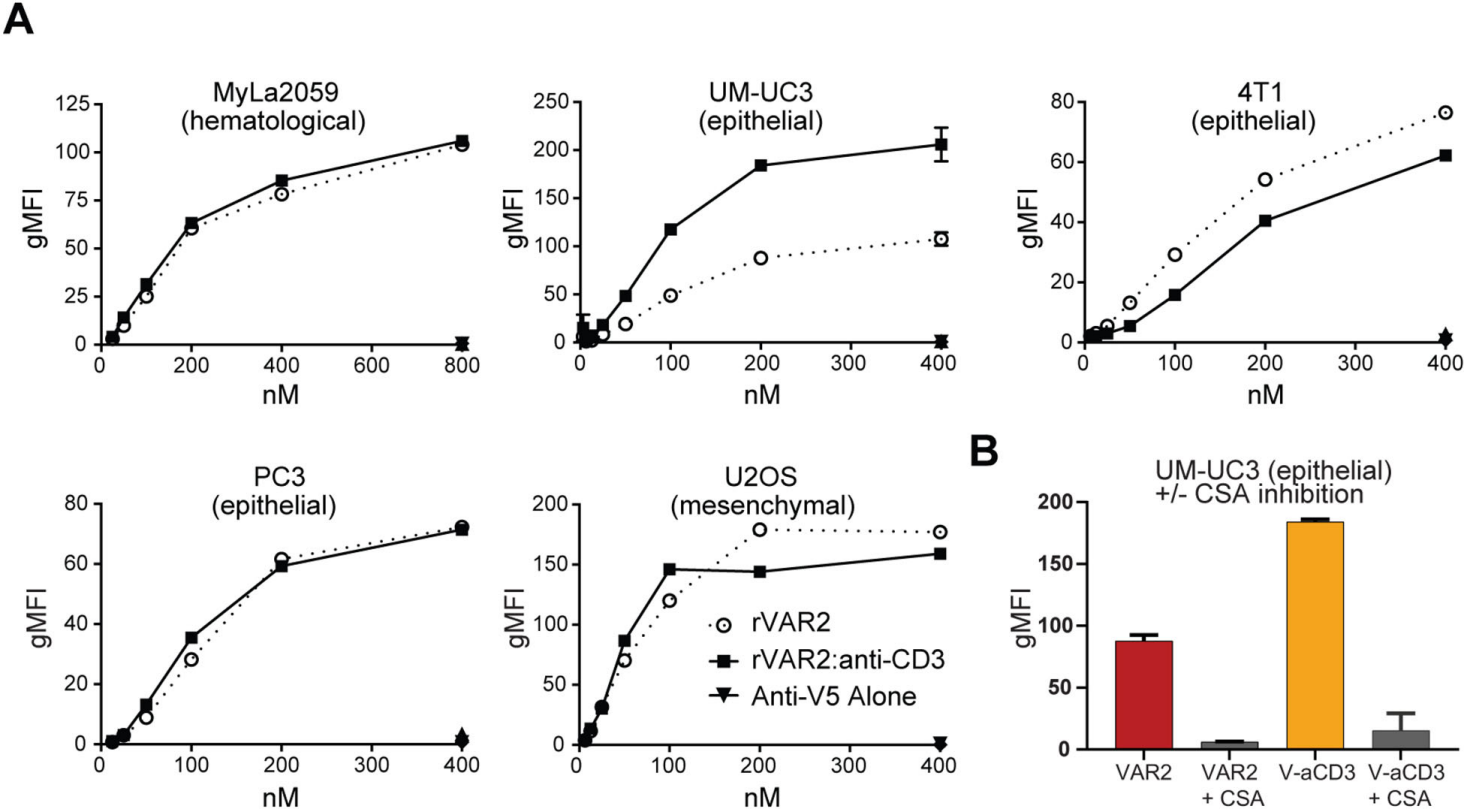
rVAR2 and V-aCD3 bind cancer cells and malignant tissue. **A** Binding was assessed by flow cytometry. Graphs represent binding based on Geometric mean fluorescence intensity (gMFI) Flow cytometer measurements of binding to various cancer cell lines of different origins. Stippled lines show binding to VAR2, solid lines show binding to V-aCD3. The V-aCD3 and rVAR2 bound to all cancer cell lines and the antibody alone did not show any binding. **B** Binding of VAR2 and V-aCD3 to cancer cells are inhibited by competition with purified CSA.

rVAR2 binds with high affinity to most cancer cells^13^. However, the interaction between ofCS and rVAR2 is complex and possibly involves a large portion of rVAR2. Therefore, conjugation of the aCD3-scFc to rVAR2 might sterically hinder binding of rVAR2 to ofCS. We thus tested binding of the V-aCD3 to five different cancer cell lines of different origin (MyLa-2059, UC-3, 4T1, PC-3, and U2OS) by flow cytometry (Fig. 2A). In four of the cell lines, V-aCD3 bound with similar level as rVAR2 alone, indicating that attachment of the aCD3-scFc did not affect rVAR2 binding to ofCS on these cells. For the UM-UC-3 (UC-3) bladder cancer cell line the binding of V-aCD3 was higher than the rVAR2 binding. Importantly, binding to cancer cells was inhibited by competition with purified CSA for both V-aCD3 and rVAR2 (Figs. 2B, S4).

### V-aCD3 induces T-cell-mediated lysis of cancer cells in vitro

**Fig. 3:**
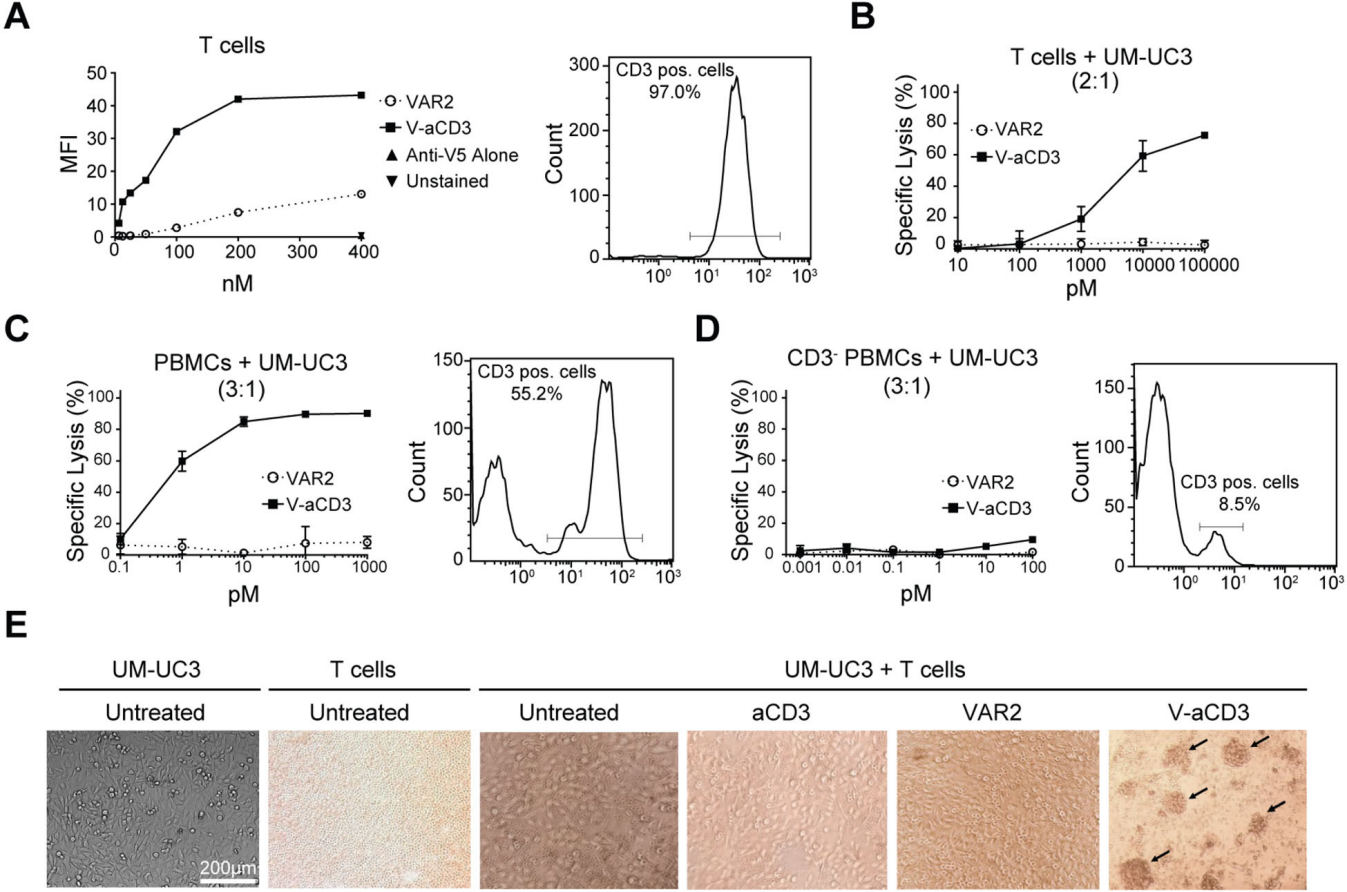
V-aCD3 bind to T cells and induces T-cell-mediated cancer cell death. **A** Binding of V-aCD3 (solid line) and of rVAR2 (dashed line) to purified T cells was assessed by flow cytometry and showed binding of V-aCD3 to T cells at low concentrations. Above 95 percent of the purified PBMCs was CD3-positive. **B** V-aCD3 mediated killing in the presence of T cells. **C** V-aCD3 mediated killing in the presence of PBMC. About 50% of the PBMC was positive for CD3. **D** V-aCD3 mediated killing in the presence of PMBC depleted of T cells. V-aCD3 (solid lines) and rVAR2 alone (dashed lines) Blood donors = 3 for each of the above experiment. Each data point represent the mean of triplicate wells. Error bars show standard error of the mean. **E** Pictures of suspensions of UC-3 cancer cells and T cells (upper panel), mixtures of these cell in the presence of cell medium, aCD3, rVAR2 or V-aCD3 (middle and lower panel). The mix of T cells and cancer cells in the presence of V-aCD3 induces cell cluster formation.

Having demonstrated that the rVAR2 component of V-aCD3 bound specifically to cancer cells, we next examined the binding properties of the aCD3 part of the bispecific V-aCD3 molecule. Using flow cytometry, we showed specific binding of the V-aCD3 to T cells from healthy donors (Fig. 3A). Additionally, we demonstrated lysis of UC-3 cells by peripheral blood mononuclear cells (PBMC), or purified CD3-positive T cells in the presence of V-aCD3, but not with rVAR2 alone (Fig. 3B, C). The cytotoxic potential of V-aCD3 was further verified on additional cancer cell lines (PC-3, U2OS) (Fig. S1). When aCD3 was tested alone (e.g., without rVAR2) it was also able to induce tumor cell killing. This may likely be caused by a propensity of the aCD3 protein to dimerize, which consequently would crosslinks receptors on the T cell leading to T-cell activation this was supported by HPLC analyses of the aCD3 protein showing a clear tendency to dimerize when not conjugated to rVAR2 (Fig. S2).

To verify the CD3 dependent activation of V-aCD3 a cytotoxicity assay was conducted in the presence of peripheral blood mononuclear cells (PBMC) or PBMC depleted of CD3-positive cells. PBMC induced specific lysis of the target cells in the presence of V-aCD3, but this was not the case for PBMC depleted of CD3-positive cells (Fig. 3C, D). Furthermore, V-aCD3 could activate T cells when co-cultured with UC-3 cells as indicated by the formation of T-cell clusters, which were not present in the presence of aCD3 or VAR2 (Fig. 3E). These experiments showed that V-aCD3 and aCD3 bound T cells and that these induced T-cell activation and subsequent killing of cancer cells in vitro. The T-cell-specific killing appeared to be augmented by the presence of the entire PBMC population possibly through engagement of immune cells through the Fc part of the aCD3 molecule.

### V-aCD3 induce cytokine release and is inhibited by anti-FASL and concanamycin A

**Fig. 4:**
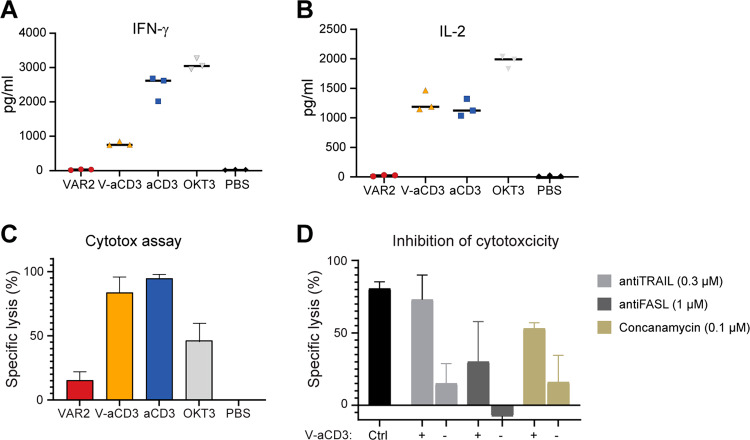
V-aCD3 mediated cytokine release and reduced cytotoxcicity by anti-FASL and concanamycin A. **A** IFN-gamma and **B** IL-2 cytokine release induced by V-aCD3 and controls. **C** Levels of tumor cell lysis (and cytokine levels) were measured after cultivation of PBMCs and UM-UC-3, effector: target (5:1) with maximum killing doses of V-aCD3 (100 nM) and controls. The data shown is three replicate measurements from one representative of three experiments using PBMC donors. Error bars represent standard error of the mean (SEM) **D** the effect of V-aCD3 activated PBMC when inhibited with anti-TRAIL, anti-FASL, and concanamycin A. PBMC was preincubated with inhibitors before applied to target cells in the presence of V-aCD3. V-aCD3 mediated killing in the presence of PBMC and inhibitors was determined by luminescence arising from oxidation of D-luciferin by luciferase expressed by viable cancer. Error bars represent SEM from triplicate determinations.

T-cell engaging immunotherapies including bispecific antibody constructs and CAR T-cell therapies have shown efficacy in several clinical settings. However, cytokine release syndrome represents one of the most frequent and serious adverse effects of these therapies^31–34^. In the case of T-cell engaging constructs, cytokine release syndrome is initiated by the extensive release of IFN-γ by activated T cells or by the tumor cells themselves. Secreted IFN-γ subsequently induces activation of other immune cells, which leads to the overproduction of other cytokines such as IL-2, IL-6, IL-10, and TNF-α^31,32^. We therefore measured IFN-γ and IL-2 production by PBMC and UC-3 tumor cells in the presence of VAR2, V-aCD3, aCD3 and a commercially available anti-CD3 monoclonal antibody (OKT3) (Fig. 4A, B). The IFN-γ was markedly higher in the presence of the anti-CD3 antibodies than in the presence of V-aCD3. VAR2 did not induce IFN-γ production. Similarly, the IL-2 production was high in the presence of OKT3 and at par in the presence of V-aCD3 or aCD3. In assays performed in parallel, tumor cell lysis was high in the presence of both V-aCD3 and aCD3 (Fig. 4C) and markedly lower in the presence of OKT3. Thus, higher levels of cytokine production did not correlate with tumor toxicity.

As a possible mechanism for V-aCD3 induced T-cell killing of cancer cells, we investigated the cytotoxic potential of V-aCD3 in the presence of concanamycin A (inhibitor of the granzyme b/perforin death pathway), anti-FASL or anti-TRAIL. While anti-TRAIL failed to inhibit V-aCD3 induced cytotoxicity, both anti-FASL and Concanamycin seemed to reduce the lytic potential of V-aCD3 (Fig. 4D). Anti-FASL reduces the lytic potential nearly 50% while concanamycin A reduces it by 25%. These data suggest that redirected lysis by V-aCD3 depends on both the Fas/FasL and perforin–granzyme pathways.

### V-aCD3 reduces tumor growth in vivo

**Fig. 5:**
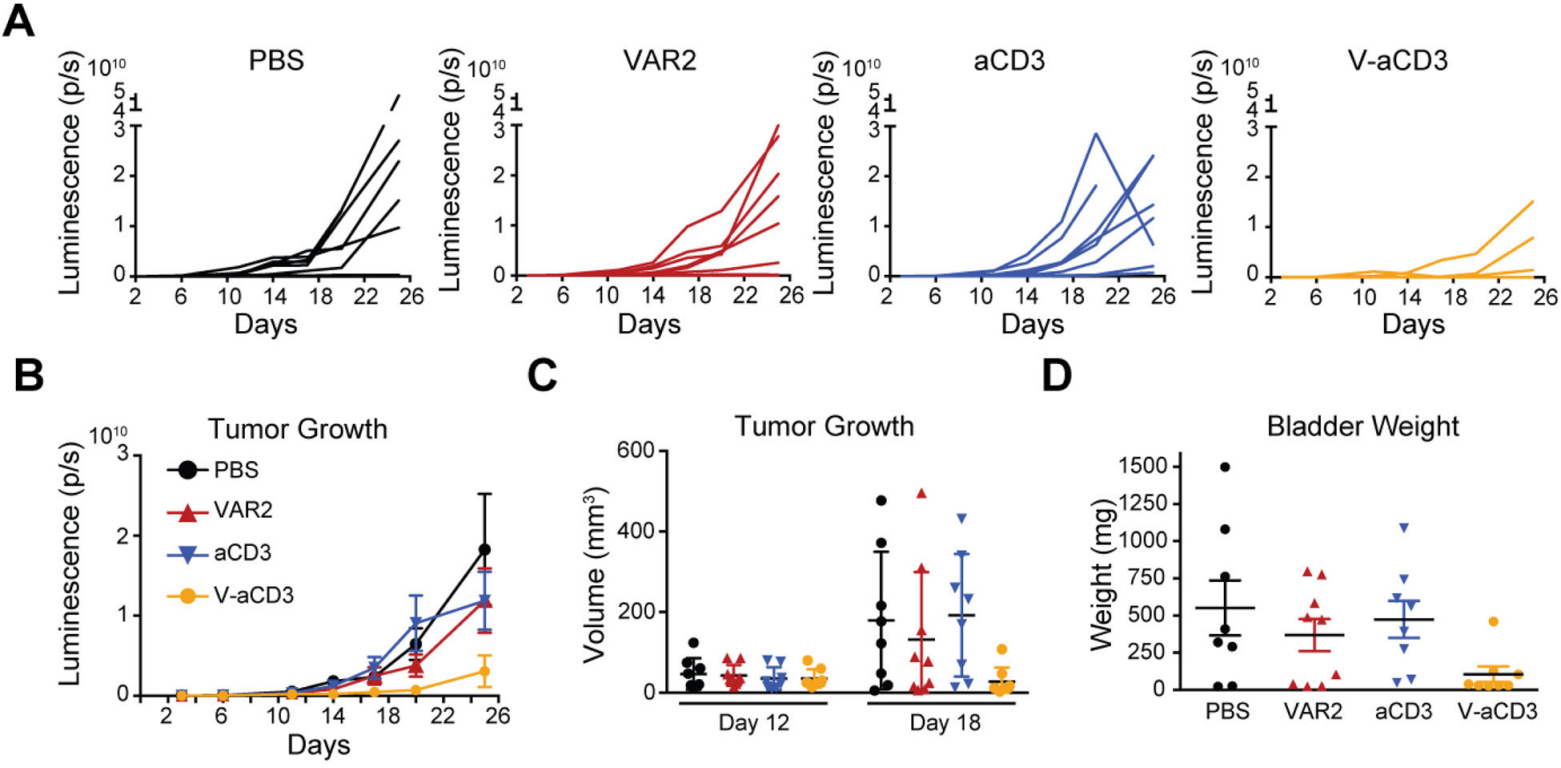
V-aCD3 impedes growth of cancer cells in vivo in an orthotopic mouse bladder cancer model. **A** Tumor size (bioluminescence signal in bladder from cancer cells). Mice were transfused with human PBMC on days 0 and 5 after tumor injection and received treatment with V-aCD3 (solid black line), aCD3-scFc (green line), rVAR2 (dashed black line), or PBS (red line). Tumor growth was statistically significantly impeded in mice treated with V-aCD3 compared to mice treated with PBS, rVAR2, or aCD3-scFc (*P* < 0.006, generalized least square model for log transformed panel data). **B** Tumor size of individual mice in the four groups, error bars are SEM of 8–9 mice. **C** Tumor size (mm3) measured by ultrasound on day 25. The tumor size of mice treated with V-aCD3 were statistically significantly lower than in mice treated with PBS or aCD3-scFc (*P* = 0.028 and *P* = 0.006, respectively, Wilcoxon test). **D** Bladder weight including the tumor volume measured when mice were sacrificed on day 25. The bladder weight of mice treated with V-aCD3 were statistically significantly lower than in mice treated with PBS or aCD3-scFc (*P* = 0.035 and *P* = 0.048, respectively, Wilcoxon test).

The anticancer potential of V-aCD3 was tested in an orthotopic xenograft model of muscle invasive bladder cancer. Luciferase-expressing UC-3 cells were injected into the bladder wall of athymic nude mice by ultrasound-guided injection as previously described^35,36^. This athymic mouse model is deficient in T cells; however, functional innate immune populations like dendritic cells, NK cells, and neutrophils remain^37^. Thus, this model is a compromise between testing a murine Fc and a human aCD3 engager, as a murine aCD3 engager was not available. Human PBMCs were co-injected, at a ratio of 2:1 (PBMC to UC-3), at the time of tumor implantation and again 5 days later. Mice were treated in the bladder wall with V-aCD3, VAR2, aCD3, or PBS at days 3, 5, 7, and 11 post-tumor implantation. Tumor growth over time was assessed by changes in bioluminescence (Fig. 5A, B). All mice had a confirmed tumor before start of treatment (Fig. S5). Tumor growth was significantly impeded in mice treated with V-aCD3 compared to mice treated with PBS, rVAR2, or aCD3 (*P* < 0.006, Generalized estimated equations model for log transformed panel data, Fig. 5B). When comparing tumor growth in individual mice, tumor growth was largely controlled in 8 of 9 mice receiving V-aCD3, whereas only a minority of mice receiving PBS, rVAR2, or aCD3 showed a similar lack of tumor growth. Comparable results were obtained whether tumor growth was assessed using bioluminescence signal of the tumor cells, size of tumor measured by ultrasonography, or weight of the bladder at the end of the study (Fig. 5B-D). Importantly, treatment with V-aCD3, but not rVAR2 or anti-CD3 alone, led to clearance of tumors in five out of eight mice (Fig. 5A) at the experimental endpoint. In addition, animals treated with V-aCD3 in the absence of PBMCs had similar tumor development as mice treated with PBMCs and PBS, and as the untreated animals (Fig. S3). These data show that V-aCD3 treatment can lead to the induction of effective antitumor immune responses in vivo.

## Discussion

The use of bispecific compounds as anticancer therapeutics is a treatment strategy with promising clinical relevance across a broad range of cancer types. Indeed, several bispecific anticancer drugs are currently approved for use in patients or are in clinical trials^3^. Anticancer bispecific compounds typically contain a moiety targeting the compound to cancer cells and an effector moiety, which directly or indirectly leads to tumor cell death. Most cancer-specific targets are proteins embedded in the cell membrane of the cancer cells^38^. A classic CD3-engaging bispecific compound would thus bind such cancer-specific target with one effector arm and a T cell through anti-CD3, bringing the two cells into close proximity, which results in the formation of an activating immunological synapse. OfCS is an uncommon cancer target since it is highly expressed in the extracellular matrix (ECM) as well as on the cell membrane^13^. The feasibility of inducing effective antitumor immune response when targeting the ECM has been demonstrated previously by bispecific antibodies targeting a tumor associated splice variant of fibronectin and CD3^39^. Therefore, ofCS is an attractive tumor target due to the omnipresence of this molecule in cancers (cell surface and/or extracellular matrix), including cancers with no available treatments. Because anti-CD3 is the effector moiety on several bispecific anticancer drugs in clinical use or under clinical development^3^, we chose to combine it with rVAR2 in an attempt to produce a bispecific molecule for anticancer immunotherapy targeting ofCS. In this proof of concept study, the bispecific molecule V-aCD3 was produced from two recombinant proteins, which were linked by forming an isopeptide bond using split protein technology^27,28^. By using distinct expression systems, we avoided compromising the optimal expression conditions for each moiety in terms of correct folding, glycosylation and production yield. In addition, separate expression allows us to test the functionality of each moiety separately and then directly compare effector functions to the conjugated molecule, hence providing true controls for V-aCD3. The binding regions of the two proteins are in theory orientated in opposite directions, enabling optimal engagement of the effector and cancer cell. The Fc domain of the anti-CD3 is a murine IgG2b which preferentially binds to FcγRI and FcγRIII receptors, but has reduced affinity toward FcγRIIB^40,41^. Therefore, V-aCD3 is ultimately a trispecific molecule that can bind target tumor cells via rVAR2, recruit and activate T cells via the anti-CD3 domain, and bind FcγR and mediate additional Fc-dependent killing capabilities, such as antibody-dependent cellular cytotoxicity (ADCC) through activation of Fc receptor-expressing cells^42,43^.

V-aCD3 bound cancer cell lines of different lineages with similar efficiency as rVAR2. The rVAR2 has previously been tested in biosensor assays and the affinity of the interaction between rVAR2 and cancer cells is high with a Kd value in the low nanomolar range^13,16^. This is similar to MEDI-365 bispecific antibody using an anti-CEA Fab fragment as targeting moiety^44^. The aCD3-scFc fragment of V-aCD3 likewise bound to effector T cells and the functionality was not compromised when fused to rVAR2 as can sometimes be the case when single-chain variable fragments (scFvs) are incorporated in immune arrangements^1^.

V-aCD3 mediated effective clustering of T cells in vitro, and this clustering resulted in cancer cell lysis by both PBMC and purified T cells. To our surprise, we found a non-rVAR2-dependent killing of cancer cells by anti-CD3. We hypothesize that although the hinge region of the scFc is excluded it may form functional dimers. This was confirmed by HPLC data (Fig. S2). When dimerized anti-CD3-scFC resemble the OKT3 antibody that is reported to stimulate T cells in vitro^45^. However, in vivo administration of OKT3 has previously been shown to induce partial depletion of T cells and unresponsiveness of CD4 T cells^46^. This is consistent with our data showing that V-aCD3 including both the VAR2 and anti-CD3 moieties impeded cancer cell growth in vivo, whereas the anti-CD3 moiety on its own did not. Interestingly, PBMCs were superior to purified T-cell populations with regard to tumor killing in vitro. In theory, the Sc-IgG2b interacts with and activates accessory cells that either express FcγRI (dendritic cells and macrophages) or FcγRIII (NK cells and macrophages)^41,47^. Activation of these accessory cells and their contact with T cells may enhance T-cell activation by providing additional co-stimulatory signals or activating cytokines and could also directly contribute to antitumor activity by phagocytosis and/or cytotoxicity. Cytokine release storm is a severe condition occurring in a subpopulation of patients receiving T-cell engaging therapies. The pathophysiology is still incompletely understood but strong T-cell activation as well as the administration route and dose of the immune engager seem to influence the risk of developing the condition^48^. We found that V-aCD3 stimulated less IFN-ƴ secretion compared to aCD3 and OKT3 alone without compromising tumor cell lysis. The high levels of IFN-ƴ induced by aCD3 could be related to a dimerization of this construct, resulting in a stronger binding of the CD3 receptor of T cells (Fig. S2). V-aCD3 might in addition reduce the risk of CSR when using intra tumor administration.

T cells can eliminate cancer cells by various mode of cytotoxic actions. A central one is the delivery of cytotoxic granule content to target cells by the formation of a cytolytic synapse. Within this synapse, subunits of the pore-forming protein perforin assemble in the target membrane creating 16 nm pores^49^. Among other proteases the acidic protease granzyme B is released into the synapse and aided by the inserted pores, induce cell death. Other mode of actions involves the expression of cell death inducing factors by the T cells. These includes the FAS ligand, TRAIL, and TNF-α. Binding to their respective cell death receptors on target cells, they induce procaspase activation and cell death^50^. Here we show that the activities of perforin/granzyme B and FAS/FASL pathway both participates in the cancer cell lysis by V-aCD3 in vitro (Fig. 4d). While stimulation via TRAIL was less likely to play a role. Many factors like the type of cancer cell, MHC expression, effector cells available, and the cancer target can influence the mode of lytic action of T cells^49,51,52^. Further experiments performed with knockout perforin, FASL or TNF-α mice could provide further information of the in vivo biological actions of V-aCD3.

After establishing the antitumor potential of V-aCD3 in vitro, we investigated its efficacy in vivo in an orthotopic bladder cancer model^36^. Bladder cancer has historically been treated with immunotherapies, such as intravesical Bacillus Calmette-Guérin (BCG) for treatment of high-grade non-muscle-invasive bladder cancer^53^, and antibodies targeting immune checkpoint molecules, such as atezolizumab, for locally advanced or metastatic urothelial carcinoma^54–59^. It is therefore a relevant tumor type for establishing the efficacy of novel immunotherapies. Here, we utilized a recently established ultrasound-guided orthotopic bladder cancer model^35,36^, using the urothelial carcinoma cell line UC-3. Since V-aCD3 does not cross-react with mouse CD3, we chose to use this xenograft model together with the addition of healthy human donor PBMC. Athymic mice with a reconstituted human immune system have been successfully employed to test immunotherapies^60–62^. V-aCD3 contains a functional murine Fc region capable of interacting with NK cells, thus the remaining immune system in the athymic mice model might supplement tumor killing, hence imitating the interplay between immune cells in a clinical setting. However, when we treated tumor bearing mice with V-aCD3 without human PBMC we did not see a significant effect on the tumor sizes compared to the controls. This indicates that the main cytotoxic effect was driven by human T cells (Fig. s3). The adoptive transfer of large numbers of PBMC into immune-deficient mice can lead to the development of graft-versus-host disease (GVHD) after a few weeks^63^. To avoid these complications, we used localized injection of a small number of PBMC directly into the bladder wall, and no signs of GVHD were observed during our study.

The xenograft model with a reconstituted human immune system allowed us to test the cytotoxic and cancer target potential of V-aCD3. To further test the cytotoxic potential of V-aCD3 it would be interesting to test the effect on established tumors. The present xenograft mice model lack a complete and fully functional immune system, and the applied PBMC is known to survive a limited period. Fast tumor growth further complicates the model. We believe that the development and use of a surrogate murine V-aCD3 in immune competent mice, will allow us to study complex therapeutic concepts that go beyond targeting the tumor. In immune competent model we will benefit from initial immune activation, rapid proliferation and immune cross talked, which are all essential functions when curing established tumors with immunotherapy. Our next step will therefore be to develop and test a murine V-aCD3 in established tumor models as well as studying the underlying biological mechanism and mode of action.

The finding that V-aCD3 distinguish between healthy and malignant tissue holds promise for a broad safety window that is particularly relevant considering the off-target toxicity documented for other bispecific antibodies like the EGFR-targeting bispecific antibodies and the chimeric monoclonal Cetuximab^64–66^. In an IVIS Spectrum CT scanner we previously showed that rVAR2 conjugated with NIR Alexa-750, localized to PC-3 and B16 tumors 10 min. post injection in tail vein^13^. The widespread expression of ofCS in cancers and the efficient targeting potential of rVAR2 offers additional advantages over bispecific molecules targeting tumor-specific antigens such as CEA, CD20, or PSMA, which are restricted to a much narrower range of cancers^67,68^.

As a malaria protein, VAR2 will to some extend be immunogenic in humans. The immunogenicity may be further enhanced by the bacterial origin SpyCatcher/SpyTag system and the murine IgG2b FC region. For clinical translation of V-aCD3, these aspects will be have to be addressed and partly avoided by expression of V-aCD3 as one genetically fused molecule and with a human FC region. On the other hand, immunogenicity can enhance the antitumor effect of bispecific antibodies, as it has been observed in a phaseII/III study with catumaxomab^69^.

Here, we have presented a proof of concept study that highlights the potential efficacy of V-aCD3 in established tumors. In order to fully explore the anticancer potential of the novel V-aCD3, further studies should include more advanced humanized animal models that recapitulate the tumor-immune interactions. Alternatively, a mouse-specific anti-CD3 can be used, which will allow for the use of immunocompetent animal models, which will enable efficacy and safety profiling that no other model can mimic.

In conclusion, we have successfully designed a bispecific molecule capable of activating effective antitumor immune responses in vitro and in vivo. Similar to rVAR2 alone, the bispecific fusion protein demonstrated binding specificity towards a wide range of tumor cell lines and had limited off-target binding to noncancer cells. This is a first proof of concept study using a recombinant pathogen derived protein as a component in a bi- or trispecific cancer immunotherapy and with further development could provide a novel cancer therapy.

## Methods and materials

### Cells and cell culture

Target cells used were the luciferase transduced PC-3 (human prostate cancer), the UC-3 (human bladder cancer) cell lines, MyLa-2059 (human T-cell non-Hodgkin lymphoma), 4T1 (murine breast cancer), and U2OS (Human Bone Osteosarcoma cells). Cell lines were kindly provided by our collaborators at Vancouver Prostate Centre, British Columbia, Canada and Clausen group, Department of Cellular and Molecular Medicine, University of Copenhagen, Denmark.

Peripheral Blood Mononuclear Cells (PBMCs) were isolated from whole blood from healthy human donors by density gradient medium (LymphoprepTM). When isolating T cells from whole blood, the blood was incubated with RosetteSepTM Human T cell Enrichment Cocktail (Stemcell, 15021) according to manufactures instructions. Depletion of T cells from isolated PBMCs were performed using RosetteSepTM Human CD3 Depletion Cocktail.

### Design and expression of the bispecific V-aCD3 molecule

Recombinant protein expression of VAR2CSA (VAR2) was carried out in *E. coli* SHuffle cells as previously described^13^. rVAR2 was expressed with an N-terminally linked SpyTag and a C-terminally linked V5 and HIS-tag. The anti-CD3 scFv-Fc molecule was constructed and produced by Fusion antibodies (Belfast, UK): The huOKT3 scFv sequence followed by a single-chain murine Fc IgG2b format with a SpyCatcher domain linked to the C-terminal. The DNA was inserted into a mammalian expression vector, transfected into CHO cells, and purified via protein G affinity chromatography followed by dialysis in PBS buffer. V-aCD3 is generated when the SpyTag on rVAR2 forms a spontaneous amide bond when binding the SpyCatcher present on the anti-CD3 molecule^29,30^. Coupling of the proteins was verified by SDS-PAGE gel.

### Flow cytometry

MyLa, UC-3, PC-3, U2OS and 4T1 cancer cells were grown to 80% confluency and harvested in an EDTA detachment solution. Cancer cells were then incubated with protein (400–3.25 nM) in PBS containing 2 % fetal bovine serum for 30 min at 4 °C. After washing, the cells were incubated with an anti-V5-FITC antibody and binding was analyzed using a FACSCalibur (BD Biosciences). A recombinant non-CS binding region of VAR2CSA (DBL4) was used as a negative control protein. Binding to CD3 was confirmed on human T cells isolated from buffy coats using EasySep™ Human T Cell Isolation Kit. V-aCD3 binding was detected with anti-V5-FITC antibody and the presence of T cells was verified by using anti-CD3-PE antibody.

### T-cell clustering

For evaluating V-aCD3 ability to activate T-cell clustering in vitro, UC-3 cancer cells were seeded into 96-well plates and allowed to adhere overnight. The following day, the medium was removed and freshly purified PBMC suspended in RPMI containing 2% FBS was added to the wells in a 10:1 effector/target ratio. V-aCD3, rVAR2 or aCD3-scFc was added to the PBMC/UC-3 cultures at a concentration of 800 nM. Cultures were incubated for 3 days, and the subsequent cluster formation was detected in the microscope.

### Cytotoxicity assay

15,000 luciferase transduced UC-3 target cells were added to each well into a black transparent 96-well flat bottom plates (Nunc). The cells was seeded overnight at 37 °C in a humidified atmosphere of 5% CO_2_. The following day, effector cells (PBMCs, purified human T cells and CD3 depleted PBMCs) was added to each well in effector target ratios 3:1 and 2:1. V-aCD3 and controls were added in a 10-fold dilution range as soluble proteins on top of the PBMCs and the plate was incubated for 48 h at 37 °C in a humidified atmosphere of 5% CO_2_. Target cells were incubated with and without effector cells to estimate background killing. Maximal killing was achieved by adding 20% DMSO (data not shown). Luminescence arising from oxidation of D-luciferin by luciferase expressed by viable cancer cells was measured in a Perkin Elmer TopCount NXT. Percentage of specific target cell lysis was calculated by the formula: Specific lysis [%] = [(Sample − PBMC background)/(Max tumor signal − PBMC background)] × 100.

### Cytokine analysis

Cytokine release was measured in vitro using 5 × 10^4^ UM-UC-3 cells, which were allowed to attach overnight. Freshly isolated PBMCs were added together with maximal efficacy treatment concentration (100 nM) of V-aCD3 and controls. Following a 24-h incubation at 37 °C, the supernatant fluid was collected and tested for IL-2 and IFNγ. The concentrations of IFNγ and IL-2 was measured using ELISA (ELISA MAX™ Deluxe Set Human IL-2/IFN-gamma, from Biolegend), following the manufacturers protocol. Briefly described, prior to running the ELISA IL-2/ IFNγ capture antibodies was added to a 96-well plate and incubated overnight (16–18 h) at 4 °C. The following day the plate wash washed and blocked with the provided blocking buffer to reduce unspecific binding for 2 h. Following a second wash, 100 μL/well of standards (IL-2 or IFNγ) or samples (supernatant diluted 1:3 in 1× Assay Diluent A) was added to the appropriate wells. The plate was incubated at RT whit shaking for 2 h. The plate was then washed and 100 ul detection antibody was added to each well, and incubated for 1 h. As a final step 100 μL of diluted Avidin-HRP solution was added to each well, and the plate was developed after 30 min. with freshly mixed TMB Substrate Solution and incubate in the dark for another 30 min. The absorbance was read at 450 nm within 15 min.

### Inhibition of cytotoxicity

Five thousands luciferase transduced UC-3 target cells were plated into a black transparent 96-well flat bottom plates (Nunc) and incubated for 18 h. In vitro generated effector cells (1 × 10^6^ PBMC/ml) were treated with 100 nM concanamycin A (ChemCruz), anti-FASL mAb (30 ug/ml) (abcamab231011), or anti-TRAIL mAb (50 ug/ml) (abcam ab2219) for 2 h. Cells were washed thoroughly before they were added at the indicated concentrations to the target cells (50.000 PBMCS/pr well). 10 nM of V-aCD3 was added to cultures containing target cells and PBMC and the plate was incubated for 24 h at 37 °C in a humidified atmosphere of 5% CO. The plate was developed as previously described for cytotoxicity assay.

### Orthotopic bladder cancer xenografts

All animal procedures were performed in agreement with Institutional Animal Care and Use Committee (IACUC). The in vivo efficacy was tested in an orthotopic bladder cancer xenograft model by percutaneous inoculation of bladder cancer cells into the anterior bladder wall as previously described^36^ 12-weeks-old athymic nude mice (Envigo) were anesthetized with 2.5% isoflurane and inoculated with 30 μL of a cell suspension in Matrigel (BD Biosciences^TM^) containing 100.000 luciferase-expressing UM-UC-3 human bladder cancer cells plus 200.000 PBMCs under ultrasound guidance as previously described. Intra/peritumoral treatments (15.4 µg V-aCD3) and controls were administrated on day 3, 5, 7, and 11. In addition a second dose of PBMCs was given in combination with treatment on day 5. Bioluminescence and ultrasound was used to monitor tumor growth. The experiment was ended at day 25, where especially the control group tumors were very large (>500 m^3^) and mice were starting to lose weight. In the orthotopic bladder models, mice cannot tolerate large tumors and get sick quickly, often because of blockage in the urinary tract or kidney failure. Bioluminescence imaging was conducted two times a week using the Xenogen In Vivo Imaging System (IVIS). Images were collected 10–20 min after i.p. injection with D-luciferin (150 mg/kg, Caliper Life Science). 3D ultrasound imaging was performed by scanning of the bladder as a whole in 0.1 mm increments and tumor volume was quantified using the Visual Sonics imaging software package.

### Statistics

Data were entered into Excel and imported into STATA15, where the statistical analyses were performed. For the in vivo experiments evaluating tumor burden in mice treated with different compounds measures of tumor burden in individual mice were declared to be panel data based on mouse id and timepoint. The effect of belonging to different treatment groups was tested in a generalized least squares regression model using the xtgls command and including treatment group and timepoints as explanatory variables. The difference between tumor burden in the different groups at day 25 was evaluated using Wilcoxon rank sum test.

## Supplementary information

Supplementary figure legends

Figure S1: In vitro Cytotoxicity

Figure S2: HPLC profiles of recombiants

Figure S3: In vivo efficacy of V-aCD3 in the absence of PBMC

Figure S4: CSA inhibition of VAR2 and V-aCD3 to target UC-3 cancer cells.

Figure S5: Bioluminescence signals pre-treatment
