# Supplementary material for: Development of a bispecific immune engager using a recombinant malaria protein
Source: Cell Death Dis. 2021 Apr 6;12(4):353. doi: 10.1038/s41419-021-03611-0 (PMC8024270; doi:10.1038/s41419-021-03611-0)
Supplement: Supplementary file 1 — Supplementary figure legends [file 41419_2021_3611_MOESM1_ESM.docx]

**Figure S1: In vitro Cytotoxicity**

Lysis of PC-3 cancer cells and U2OS by PMBCs in the presence of V-aCD3 (solid lines), VAR2 alone (dashed lines) or aCD3-scFV (blue line). Concentration ranging from 10000 pM to 0.1 pM.PBMC donor N=3.

**Figure S2**: **HPLC profiles of recombiants**

High pressure liquid chromatography was performed on the Agilent 1100 series system, consisting of a Binary pump, 96 well-plate autosampler, thermostatic column compartment and Agilent 1100 Series diode-array detector (Agilent Technologies, Denmark). Absorbance of the compounds was monitored at280 nm using a reference wavelength of 360 nm. Data acquisition was performed using Agilent ChemStation Software. The compounds were analysed on a Size exclusion column (Superdex 200 Increase, GE Healthcare). Column temperature was adjusted to 25 °C. The flow rate of the mobile phase was set at 0.075 ml/min.

The panel demonstrates the HPLC elution profiles of rVAR2 (green line), aCD3-scFV (Blue line) and V-aCD3 (Purple line).

**Figure S3: *In vivo* efficacy of V-aCD3 in the absence of PBMC**

Tumor size of individual mice treated with **a** PBS or **b** V-aCD3 in the absence of human PBMC.

**Figure S4: CSA inhibition of VAR2 and V-aCD3 to target UC3 cancer cells.**

Binding of VAR2 and V-aCD3 to cancer cells are inhibited by competition with purified soluble CSA.

**Figure S5: Bioluminescence signals pre-treatment**

Tumor size (bioluminescence signal in bladder from cancer cells) on day 3 (pre-treatment) for each individual mice. The mice without a significant tumor signal was excluded from the study.
