# Supplementary figures and images for: Development of a bispecific immune engager using a recombinant malaria protein
Source: Cell Death Dis. 2021 Apr 6;12(4):353. doi: 10.1038/s41419-021-03611-0 (PMC8024270; doi:10.1038/s41419-021-03611-0)

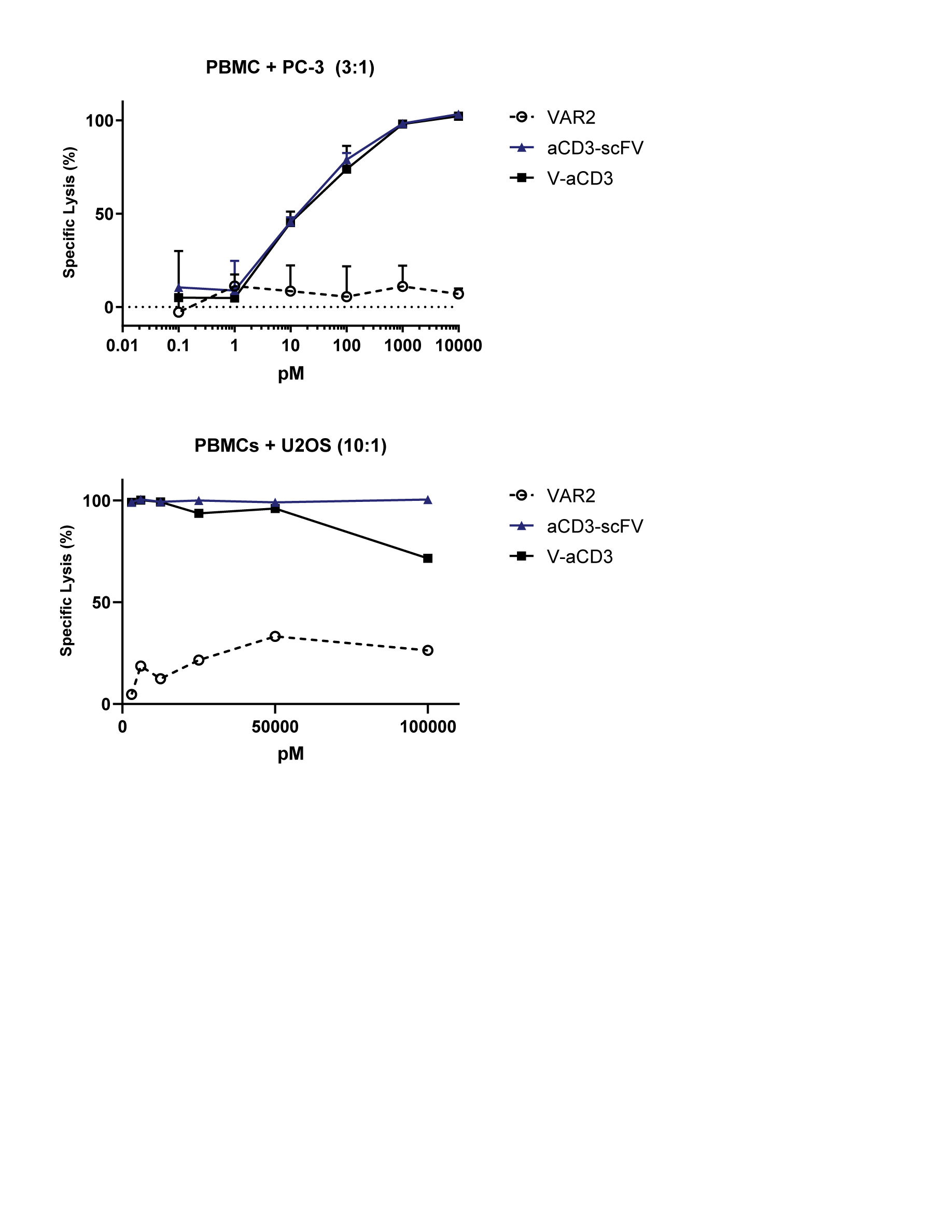

Supplement: Supplementary file 2 — Figure S1: In vitro Cytotoxicity [file 41419_2021_3611_MOESM2_ESM.jpg]

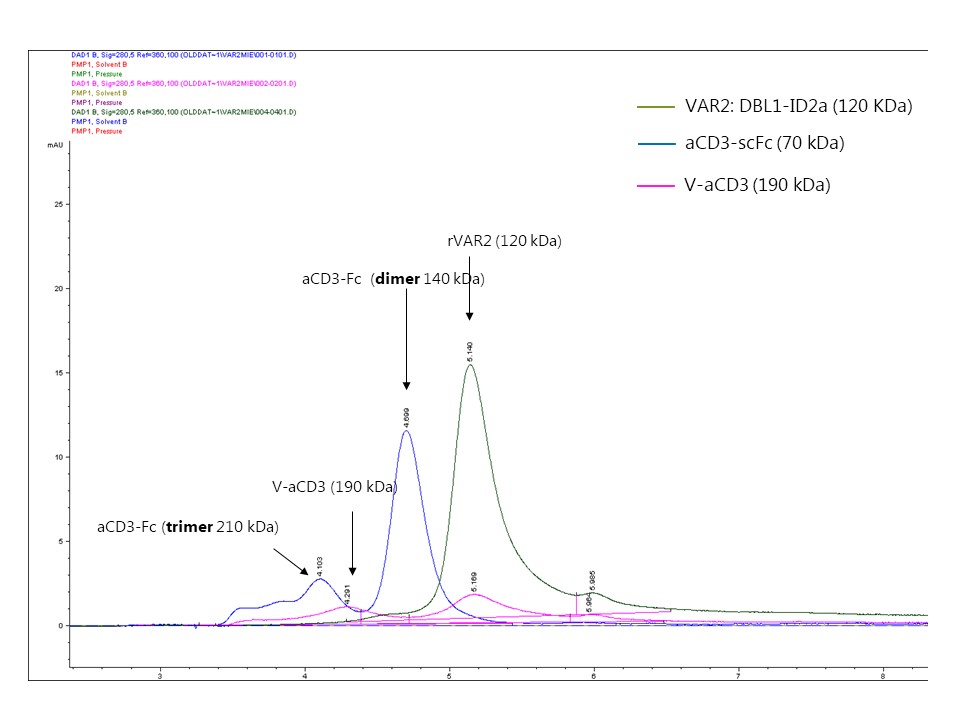

Supplement: Supplementary file 3 — Figure S2: HPLC profiles of recombiants [file 41419_2021_3611_MOESM3_ESM.jpg]

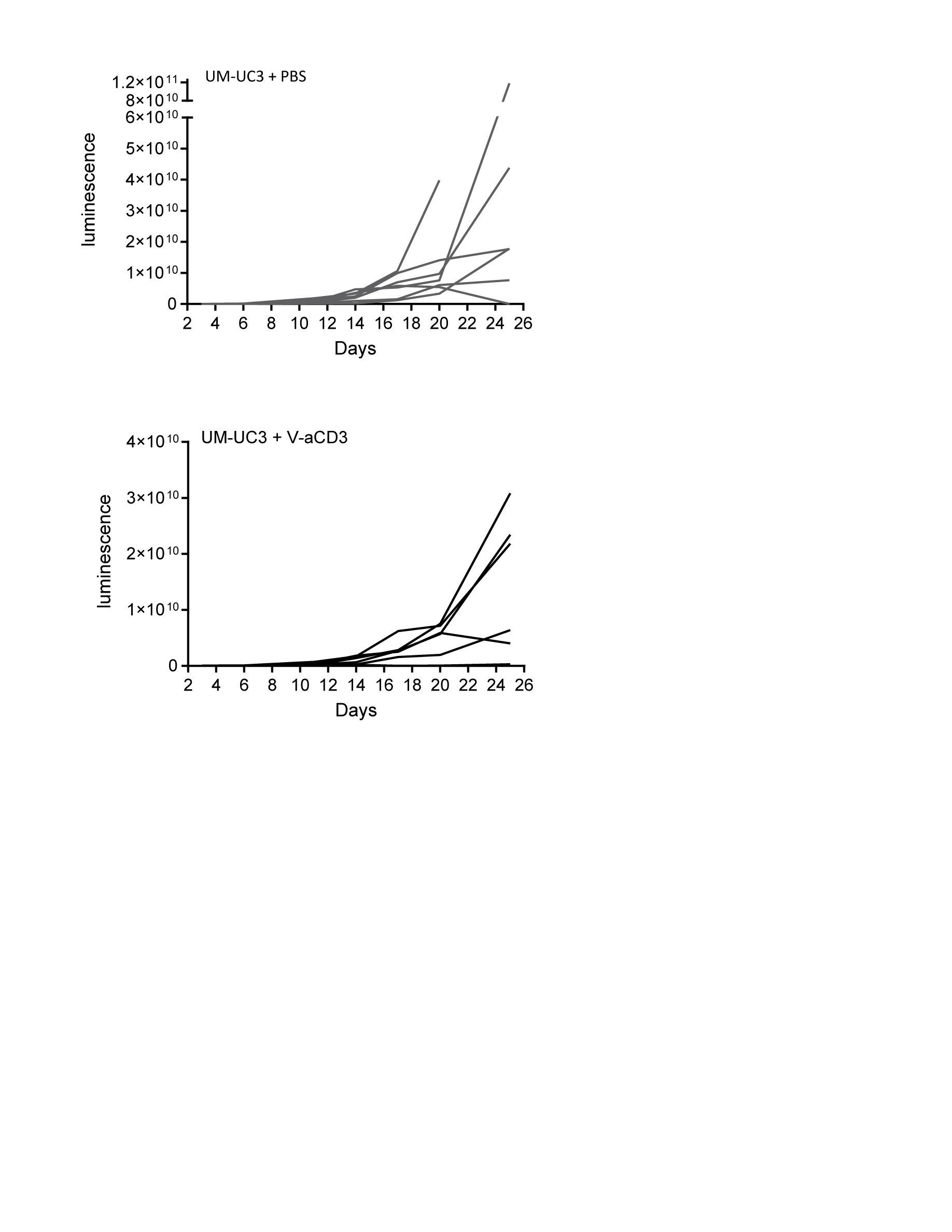

Supplement: Supplementary file 4 — Figure S3: In vivo efficacy of V-aCD3 in the absence of PBMC [file 41419_2021_3611_MOESM4_ESM.jpg]

Figure S4

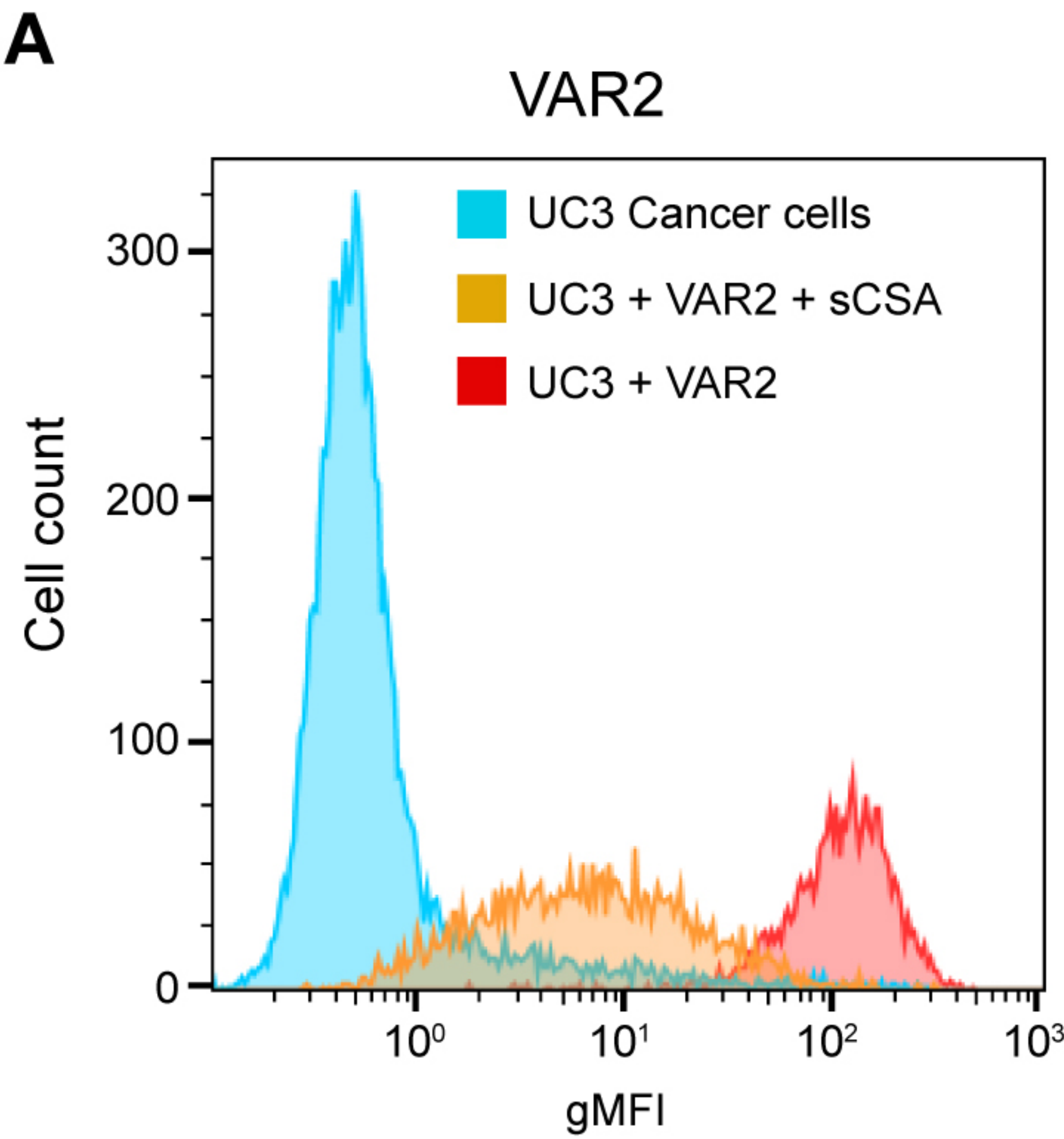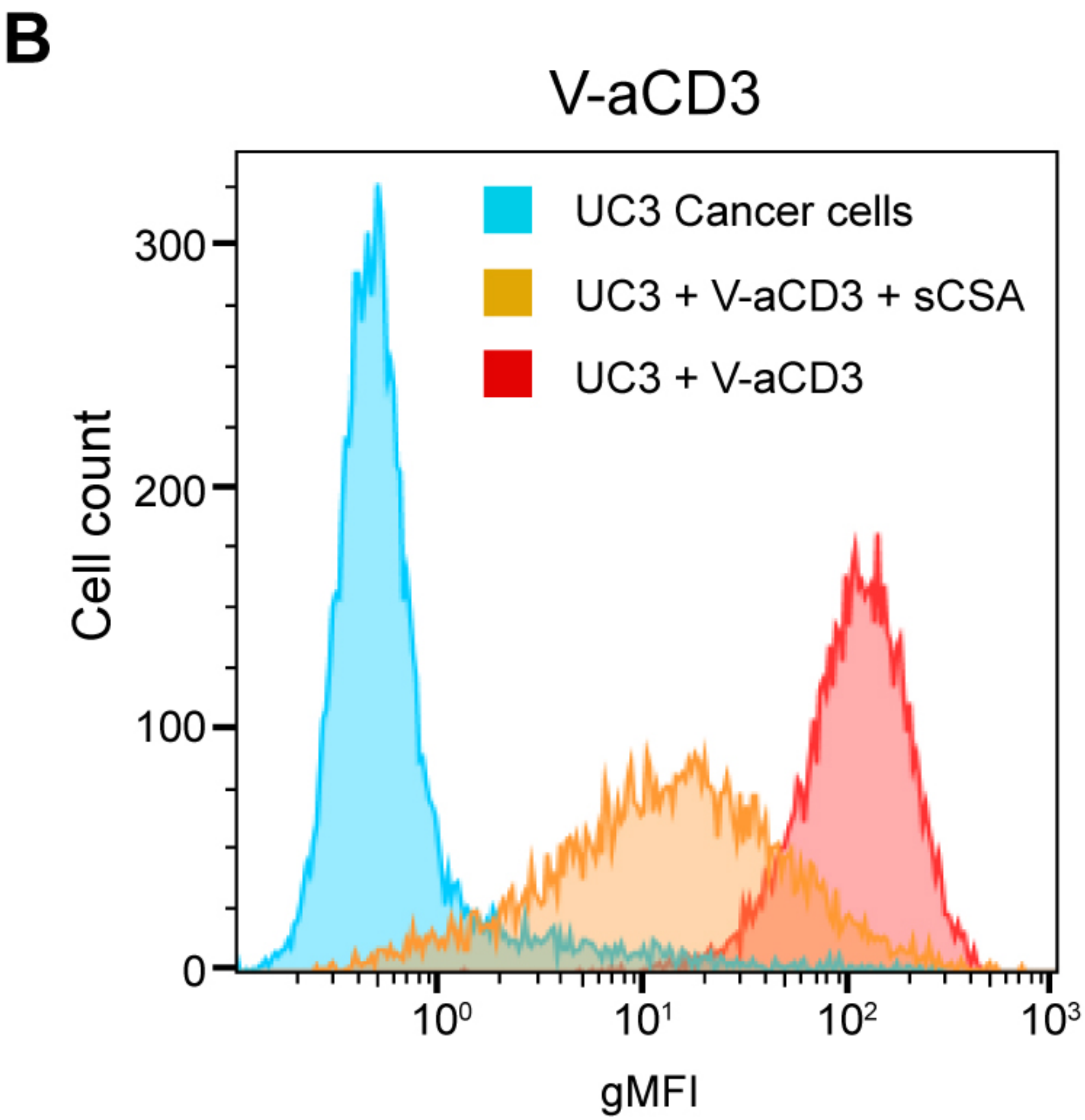

Supplement: Supplementary file 5 — Figure S4: CSA inhibition of VAR2 and V-aCD3 to target UC-3 cancer cells. [file 41419_2021_3611_MOESM5_ESM.pdf]
