# Supplementary material for: Development of a bispecific immune engager using a recombinant malaria protein
Source: Cell Death Dis. 2021 Apr 6;12(4):353. doi: 10.1038/s41419-021-03611-0 (PMC8024270; doi:10.1038/s41419-021-03611-0)
Supplement: Supplementary file 6 — Figure S5: Bioluminescence signals pre-treatment [file 41419_2021_3611_MOESM6_ESM.pdf]

Figure S5

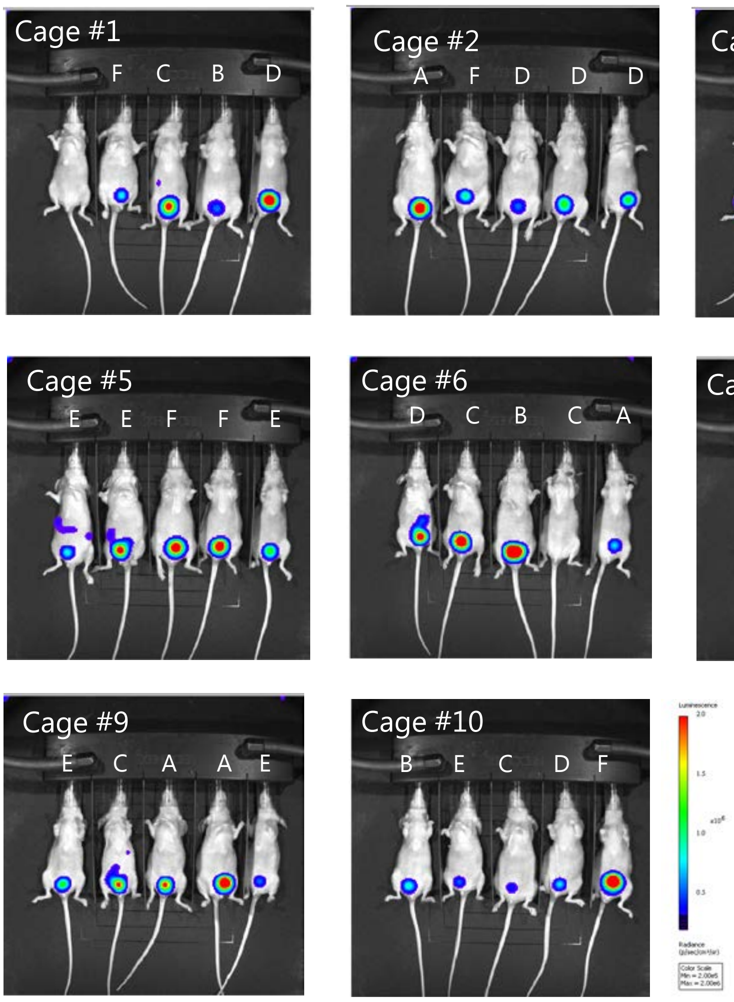

Cage #1	Group	Cage #2	Group	Cage #3	Group	Cage #4	Group	Cage #5	Group
6,15E+04	not used	2,75E+07	A	4,07E+07	F	5,55E+06	F	1,40E+07	E
9,43E+06	F	9,27E+06	F	1,71E+07	A	1,16E+07	F	2,60E+07	E
3,25E+07	C	6,85E+06	D	1,97E+07	B	2,54E+07	E	3,44E+07	F
9,81E+06	B	1,18E+07	D	1,35E+07	C	2,48E+07	E	3,24E+07	F
4,01E+07	D	1,06E+07	D	9,81E+06	D	3,49E+07	B	1,30E+07	E
Cage #6	Group	Cage #7	Group	Cage #8	Group	Cage #9	Group	Cage #10	Group
2,78E+07	D	1,33E+07	C	3,92E+07	B	1,25E+07	E	2,15E+07	B
4,12E+07	C	1,17E+07	C	2,84E+07	D	2,24E+07	C	6,17E+06	E
4,13E+07	B	2,65E+07	F	2,34E+07	C	2,06E+07	A	5,54E+06	C
1,93E+06	C	2,92E+07	A	2,47E+07	D	4,02E+07	A	8,64E+06	D
9,17E+06	A	2,18E+07	C	5,47E+07	A	7,04E+06	E	6,16E+07	F

Treatment Groups: A) PBS, B) PBMC + PBS, C) PBMC + VAR2, D) PBMC+aCD3-scFC, E) PBMC + V-aCD3, F) V-aCD3
